# Distributed attention beats the down-side of statistical context learning in visual search

**DOI:** 10.1167/jov.20.7.4

**Published:** 2020-07-06

**Authors:** Artyom Zinchenko, Markus Conci, Johannes Hauser, Hermann J. Müller, Thomas Geyer

**Affiliations:** Department Psychologie, Ludwig-Maximilians-Universität München, Munich, Germany; Department Psychologie, Ludwig-Maximilians-Universität München, Munich, Germany; Department Psychologie, Ludwig-Maximilians-Universität München, Munich, Germany; Department Psychologie, Ludwig-Maximilians-Universität München, Munich, Germany; Department Psychologie, Ludwig-Maximilians-Universität München, Munich, Germany

**Keywords:** attention, visual search, contextual cueing, adaptation, statistical learning

## Abstract

Spatial attention can be deployed with a narrower focus to process individual items or distributed relatively broadly to process larger parts of a scene. This study investigated how focused- versus distributed-attention modes contribute to the adaptation of context-based memories that guide visual search. In two experiments, participants were either required to fixate the screen center and use peripheral vision for search (“distributed attention”), or they could freely move their eyes, enabling serial scanning of the search array (“focused attention”). Both experiments consisted of an initial learning phase and a subsequent test phase. During learning, participants searched for targets presented either among repeated (invariant) or nonrepeated (randomly generated) spatial layouts of distractor items. Prior research showed that repeated encounters of invariant display arrangements lead to long-term context memory about these arrays, which can then come to guide search (contextual-cueing effect). The crucial manipulation in the test phase was a change of the target location within an otherwise constant distractor layout, which has previously been shown to abolish the cueing effect. The current results replicated these findings, although importantly only when attention was focused. By contrast, with distributed attention, the cueing effect recovered rapidly and attained a level comparable to the initial effect (before the target location change). This indicates that contextual cueing can adapt more easily when attention is distributed, likely because a broad attentional set facilitates the flexible updating of global (distractor-distractor), as compared to more local (distractor-target), context representations—allowing local changes to be incorporated more readily.

## Introduction

The visual system has an exceptional ability to extract invariances and regularities from the environment and use this information to guide visual attention. For instance, search for a particular target object can be facilitated by its surrounding, familiar context. For example, a mailbox can be identified more quickly in a front yard as compared to a kitchen environment, demonstrating that past experience can optimize visual object recognition (Palmer, 1975; see also Biederman, 1972; Davenport & Potter, 2004; Conci & Müller, 2014). A related finding in visual search tasks has been termed *contextual cueing* (Chun & Jiang, 1998), referring to improved search performance for targets presented within invariant (“repeated”) distractor configurations. In the typical contextual-cueing paradigm, participants search for a target letter “T” presented among a set of distractor letters “L.” Unbeknownst to participants, half of the trials contain repeated spatial arrangements of distractor items and the target, whereas the other half of trials presents a randomly compiled arrangement of search elements. A common finding is that over the course of the experiment, reaction times (RTs) to the target come to be speeded for repeatedly encountered relative to non-repeated displays, while participants are usually unable to discriminate repeated from nonrepeated displays above chance level. This pattern has been taken to suggest that invariant spatial target-distractor relations are extracted and stored in implicit (long-term) memory, guiding, or “cueing,” search to the target location when re-encountering a repeated display (which acts as an effective retrieval cue). This proposal receives support, for instance, from electrophysiological investigations showing that the repeated context modulates an event-related potential commonly thought to index the allocation of spatial attention to the target item (the so-called N2pc; Johnson, Woodman, Braun, & Luck, 2007). In line with this, eye-tracking studies have revealed that the incidence of first fixations being allocated to the target is also higher for repeated (vs. non-repeated) contexts (Manginelli & Pollmann, 2009; Jiang, Won, & Swallow, 2014; Peterson & Kramer, 2001). Together, these findings indicate that invariant context layouts can facilitate search by improving the guidance of focal attention to the target location (see also Geyer, Müller, & Zehetleitner, 2010).

Whereas contextual cueing demonstrates that the learning of statistical regularities provides a powerful means for facilitating search guidance in familiar contexts, the updating of previously acquired context memories has been shown to be far less efficient and rather inflexible (Manginelli & Pollmann, 2009; Makovski & Jiang, 2010; Conci, Sun, & Müller, 2011; Zellin, Conci, von Mühlenen, & Müller, 2013a; Annac, Conci, Müller, Geyer, 2017). For instance, Zellin, von Mühlenen, Müller, and Conci (2014) had their observers perform a search task that was divided into a learning and test phase. In both phases, half of the trials contained a target presented within an invariant spatial layout of distractor items, allowing participants to learn contextual associations of the constant target position relative to the constant distractor locations. Contextual cueing was found to emerge relatively rapidly, after some 3-6 repetitions of each repeated display, in line with Chun and Jiang's (1998) original findings. However, changes of the target location (while leaving the distractor locations unchanged) at the transition from the training to the test phase completely abolished the contextual-cueing effect and the gains derived from repeated (i.e., after the target location change again constant) search contexts recovered only slowly with extended training on the relocated displays. It thus appears that while initial context learning is rather quick and efficient, the effects of the initial learning interfere with the subsequent updating of already established target-distractor associations (Zinchenko, Conci, Taylor, Müller, & Geyer, 2019). We refer to this as the “down-side” of spatial context learning.

One idea of how this may work is that in learnt displays (that give rise to contextual cueing), the target location is pinpointed relatively quickly, perhaps within the first 100 milliseconds (ms) post display onset (Chaumon, Drouet, & Tallon-Baudry, 2008)—owing to the search display “automatically” retrieving the appropriate (acquired) memory representation, within which the target location is prioritized thus facilitating attentional selection (indexed by the N2pc wave; Johnson et al., 2007). However, this automatic (search-display-to-context-memory) matching process turns out to be wrong after a change of the target location (i.e., during the adaptation phase), in which case the memory “prior” mis-guides attention to, the initially learnt but now incorrect item location (Manginelli & Pollmann, 2009).

Of note, there is evidence that it is the local context of distractor items in the direct vicinity of the target, rather than the global context of the display as a whole, that is the main source of the contextual-cueing effect (see Brady & Chun, 2007; Shi, Zang, Jia, Geyer, Müller, 2013). This may be related to the fact that contextual cueing is typically investigated using a relatively hard letter search task (see above) which requires a narrow focus of attention. Given that associations of the constant target position with the invariant distractor locations are formed, or strengthened, upon detection of the target on a given trial (Ogawa & Watanabe, 2010), it is therefore not surprising that observers develop a relatively narrow, *local* context representation. This local representation incorporates multiple associations between the target and each individual distractor item in its surround (Brady & Chun, 2007), where each (additional) local distractor will effectively increase the conspicuity of the target within its item surround. Consequently, changes of the target location would produce strong interference because the local (inter-)item associations acquired during initial learning would continue to (mis-)guide search toward the initial target position.

These considerations concerning the imperviousness of established context memories to adapt to (and incorporate) changes of the target location raise an intriguing question: would a manipulation of observers’ visual scanning mode from being more narrowly “focused” to being more widely “distributed” influence their ability to adapt their existing contextual representations to relocated targets? For instance, Treisman (2006) showed that visual attention may either be widely *distributed*, spreading across an entire scene, or narrowly *focused* on only a single object at a given time (see also LaBerge & Brown, 1989; Srinivasan, Srivastava, Lohani, & Baijal, 2009). A focused attentional mode is typically associated with “serial” scanning of individual items, with an attentional spotlight illuminating only a relatively small region of the display (Posner, 1980; Treisman & Gelade, 1980), hampering the extraction of more broadly spaced inter-item relations. By contrast, a wider attentional focus, would—according to Treisman—facilitate the detection of statistical regularities in the visual displays (Chong & Treisman, 2003; Chong, Joo, Emmanouil, & Treisman, 2008; Treisman, 2006), which can subsequently come to inform search guidance (see Wolfe, Vo, Evans, & Greene, 2011, for review). The type of distributed vs. focused attention mode may be particularly effective during context adaptation, e.g., because representations of the wider context formed during initial learning (with distributed scanning) can be rapidly associated with the changed target position (e.g., Beesley, Vadillo, Pearson, & Shanks, 2015).

The aim of this study was to examine whether making observers adopt a more distributed, as compared to a more focused setting of the attentional window would help the adaptation of contextual cueing to relocated targets. In particular, we asked participants to keep fixating the center of the screen—and thus use peripheral vision, that is, adopt a distributed-attention mode for performing the search task. We contrasted this condition with a standard, focused-attention mode in which observers were free to move their eyes and scrutinize the narrower display region to find the target (for a similar approach see, e.g., van Asselen & Castelo-Branco, 2009; Higuchi & Saiki, 2017; Makovski & Jiang, 2011). Previous eye-tracking studies of contextual cueing have shown that participants require fewer fixations to find the target in repeated, as compared to randomly arranged, search arrays (Manelis & Reder, 2012; Manginelli & Pollmann 2009; Peterson & Kramer 2001; Tseng & Li 2004). Moreover, Tseng and Li (2004; see also Kröll, Schlagbauer, Zinchenko, Müller, & Geyer, 2019) observed that the RT benefit for repeated contexts is correlated with a reduction in the number of eye fixations, indicating that acquiring a memory of the invariant layout of the search array effectively shortens the scan-path that the eye takes to reach the target location.

We predicted that distributed (vs. focused) attention would lead to more efficient context adaptation, because distributed attention should allow observers to represent a larger region of the search display that would, in turn, permit the changed target position (in the unchanged overall-display arrangement) to be incorporated more readily into the existing memory representation. Importantly, the “distributed” search condition (with the eye fixed) may involve a series of covert shifts of the “attentional spotlight” to find the target, but one would nevertheless have to assume that the spotlight would, on average encompass a larger subregion of the search display in peripheral vision, as compared with the “focused” search condition (in which the eyes are allowed to fixate freely on any subregion). This would be so given that visual acuity declines, and receptive-field sizes (which ultimately determine spotlight size) increase, toward the periphery—thus impacting the resolution of local interitem relations (cf. Carrasco, Evert, Chang, & Katz, 1995). Accordingly, we predicted that the distributed condition would engender a broader attentional set that promotes the build-up of more global (distractor-distractor) context representations, as compared with the focused condition promoting the acquisition of more local (distractor-target) representations.

## Methods

### Participants

Fifty-two participants took part in the study (36 female, 3 left-handed, mean age = 26.7, *SD* = 2.8, range = 19–34). Each participant was randomly assigned to either the distributed (N = 26) or the focused (N = 26) attention condition. Data of one participant from the focused condition was lost due to a computer problem during data acquisition. Additionally, the data from three participants from the distributed and two participants from the focused conditions were removed due to a high number of error rates (>15%; > 3 S*D*s from the mean error rate). Accordingly, the data analyses reported below are based on a sample of 23 participants per distributed and focused attention condition.

The sample size was determined on the basis of previous comparable studies (e.g., Assumpção, Shi, Zang, Müller, & Geyer, 2015; Geringswald, Herbik, Hofmüller, Hoffmann, & Pollmann, 2015; Geyer et al., 2010; Zellin et al., 2011; Zellin et al., 2013a; Zellin von Mühlenen, Müller, & Conci, 2013b; Zellin von Mühlenen, Müller, & Conci, 2014; Zinchenko et al., 2018), which typically tested around 14 (or fewer) participants. Sample size estimation was informed by previous contextual-cueing studies using a training-phase/test-phase design (e.g., Assumpção et al., 2015; Geringswald et al., 2015; Zellin, von Mühlenen, et al., 2013b; Zellin et al., 2014; Zinchenko et al., 2019). On the basis of the number of participants tested in and statistical measures provided by these studies, a sample size of 12 to 14 participants suffices to detect a lack-of-adaptation effect with a power of 0.8 in a single experiment. Thus, on the basis of the studies mentioned above, one would expect the cueing effect to vanish after the target-location change, which would be evidenced by a significant two-way context by phase interaction. In this study, we compared two separate groups (distributed vs. focused attention), and a difference in contextual-cueing adaptation between the two attention modes should then be reflected by a 3-way interaction. To test this previously not reported interaction effect involving the between-subjects factor search mode, we quadrupled our sample size to 52 observers (assuming that the novel, three-way interaction is about half the size of the previously reported lack-of-adaptation, i.e., context x phase interaction, effect, which would require 4 × 13 = 52 observers). We also conducted a replication experiment with restricted viewing (see General Discussion Section). For this (replication) experiment, the relevant sample-size calculation measures (that achieve power of 80%) were taken from the actual distributed-attention condition in the main experiment.

### Apparatus and stimuli

**Figure 1.**
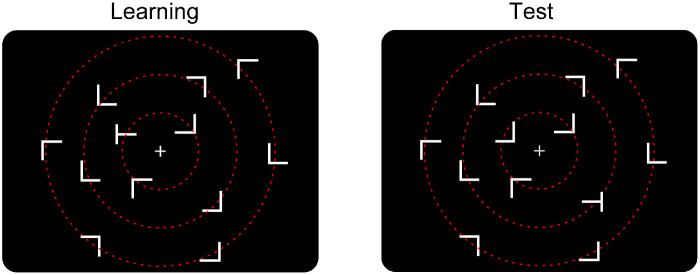
Example search display presenting a repeated target-distractor configuration. In the display, the target position swaps with a distractor from the opposite hemifield across the learning and test phases, while all other items remain unchanged—so as to examine how the target location change affects the RT advantage for repeated versus nonrepeated displays (the contextual-cueing effect) when visual search is performed under distributed—versus focused-attention conditions. Note that the red, dashed circles, depicting the three concentric rings on which the search items were arranged, were not shown in the actual search displays. In the distributed attention condition, no fixation cross was shown in the actual search displays.

The experimental routine was programmed in Matlab with Psychtoolbox extensions (Brainard, 1997; Pelli, 1997) and was run on an Intel PC under the Windows 7 operating system. Participants were seated in a dimly lit booth in front of a 19-inch CRT monitor (AOC, Amsterdam; display resolution 1024 × 768 pixels; refresh rate: 85 Hz) at a viewing distance of 60 cm (controlled by a chin rest). The search displays consisted of 12 grey items (luminance: 1.0 cd/m^2^; 1 target and 11 distractors) presented against a black background (0.11 cd/m^2^). All stimuli extended 0.35° of visual angle in both width and height. As depicted in Figure 1, the items were arranged on three (invisible) concentric circles around the display center (with a radius of 1.74°, 3.48°, and 5.22° for circles 1 through 3, respectively). In *repeated* displays, the location of the target and the location and identities (i.e., orientations) of distractors were held constant across trials. In *nonrepeated* displays, all distractors were generated anew on each trial. There were overall 24 possible target locations, eight of which were used for *repeated* displays with constant distractor layouts in the learning phase. Another eight target locations were used for *nonrepeated* displays with random distractor arrangements. And another set of eight target locations was used for repeated displays in the test phase. In the latter, the target item was always swapped with one of the distractors in the opposite hemifield (see Figure 1). For each set of target locations per condition (repeated displays in learning; nonrepeated displays in learning; repeated displays in test), there were two targets presented in each of the four display quadrants. Amongst the eight targets, two appeared on circle 1 and three other targets were presented on circles 2 and 3 each. Importantly, participants were not informed about the fact that some of the search arrays were presented repeatedly, and they were not told about the target location swap in the middle of the experiment. The “T” target was rotated randomly by 90° to either the left or the right. The 11 remaining items were L-shaped distractors rotated randomly at orthogonal orientations (0°, 90°, 180°, or 270°). Figure 1 presents example display layouts. Note that repeated search arrays were generated randomly for each participant at the beginning of the search task. We also controlled for the distance of the target from to the display center (rings 1–3), as well as the quadrant in which it was placed (see details above). The same constraints relating to the positioning of the target also applied to the way nonrepeated displays were generated, thus mitigating effects of target probability cueing (e.g., Geng & Behrmann, 2005), except that these display arrangements were, by definition, never repeated. It is thus unlikely that specific, low-level display features (uniquely) relating to the spatial composition of repeated displays had a systematic influence on contextual learning and adaptation in our experimental conditions.

To maximize the amount of trials without any eye movements in the distributed-attention condition, a video-based eye-tracker was used to monitor eye movements online (EyeLink 1000; SR Research Ltd., Mississauga, Ontario, Canada; version 4.594). If participants moved their gaze more than 1.5° away from the central fixation cross (1.7% of all trials), a beep was played to serve as a warning signal. Eye movements were also recorded in the focused-attention condition, in which participants were free to move their eyes while searching for the target. Eye-movement recordings were calibrated at the start of the experiment and after every four blocks (of 64 trials). Calibration was considered accurate when fixation positions fell within ∼1.0° for all calibration points. The default psychophysical sample configuration of the eye-tracking system (i.e., saccade velocity threshold set at 35°/s, saccade acceleration threshold set at 9500°/s^2^) was adopted for the eye-data samples.

### Trial sequence

A trial started with the presentation of a central fixation cross (0.10° × 0.10°, luminance: 1.0 cd/m^2^) for 500 ms. Next, in the focused-attention condition, the fixation cross was removed from the screen and a blank interval was presented for 200 ms, after which the search display was presented. In the distributed-attention condition, the sequence of displays in a given trial was the same, except that the fixation cross remained present on the screen throughout a given trial. Observers were instructed to respond as quickly and accurately as possible to the orientation of the target “T” (left vs. right) and either move their eyes freely (focused-attention task) or maintain fixation on the central cross while performing the visual search task using peripheral vision (distributed-attention task). Each search display stayed on the screen until a manual response was elicited. If the “T” was rotated to the right (left), observers responded by pressing the right (left) arrow button on a computer keyboard with their right (left) index finger. Following a response error, the word “Wrong” appeared on the screen for 1000 ms. Each trial was followed by a blank intertrial interval of 1000 ms. The learning and test phases consisted of 256 trial each (16 blocks × 16 trials each, 50% repeated displays in each block), with a 5-minute break in between the two phases. Participants were free to continue with the next block at their own pace. The experiment took approximately 50 minutes to complete.

### Recognition test

At the end of the experiment (i.e., after the last block in the test phase), observers performed a yes/no (repeated/non-repeated) recognition test, permitting us to assess whether they had acquired any explicit memory of the repeated configurations presented in the preceding search task of the experiment (a “standard” procedure in contextual cueing experiments; cf. Chun & Jiang, 1998). To this end, observers were presented with eight repeated displays from the initial learning phase (in which the target item was present in its original position) and eight newly composed displays. The task was to indicate whether a given display was shown previously by pressing the left or the right mouse button, respectively. The eight repeated and the eight newly generated displays were presented in random order for two times (in two separate blocks), yielding a total of 32 recognition trials, to increase the statistical power of the forced-choice recognition test (cf. Vadillo, Konstantinidis, & Shanks, 2016). Nonrepeated displays were also presented two times to equate repetitions across the previously presented and the baseline (foil) displays. Observers’ responses in the recognition task were nonspeeded, and no error feedback was provided.

## Results

For the RT analyses, error trials and “extreme” RTs less than 200 ms and more than 2000 ms were excluded from the data. This outlier criterion led to the removal of ∼5% of all trials. Individual observer's mean RTs, and associated error rates, were calculated per experimental condition and submitted to a 2 × 2 × 4 × 2 mixed-design analysis of variance (ANOVA) with the within-subject factors phase (learning, test), context (repeated, nonrepeated configurations), and epoch (one to four in learning and five to eight in test, where one epoch consists of four consecutive blocks of 16 trials each), and the between-subject factor search mode (distributed, focused attention). Greenhouse-Geisser corrected values are reported in case Mauchley's test of sphericity was significant (*p* < 0.05). In case of significant interactions, Bonferroni-corrected pairwise *t*-tests were used for further comparisons. Data analyses were performed with R version 3.4.3 (R Core Team, 2018).

Overall, observers were highly accurate in performing the search task, with an average error rate of ∼ 3%, and without an indication of a speed-accuracy trade-off. In the error rates, no main or interaction effects reached significance (all *p*s > 0.05).

### Reaction times

**Figure 2.**
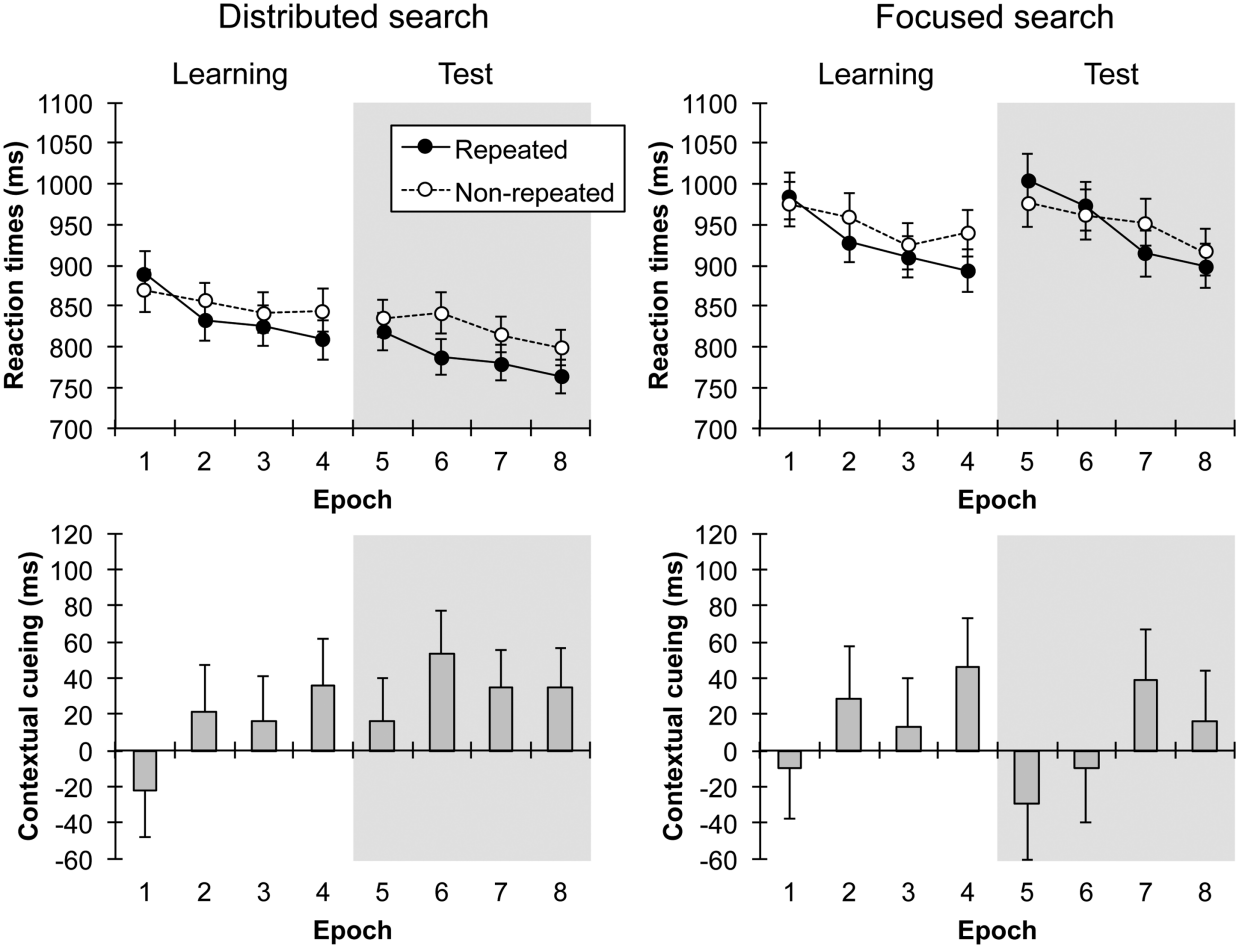
Distributed and focused attention conditions (left and right panels, respectively). The upper panels of the figure depict mean RTs (in ms) and associated standard errors for repeated and non-repeated displays as a function of epoch in the learning and test phase. The panels in the lower half represent the corresponding mean contextual-cueing effects (in ms) in each epoch.

The analysis of the RTs revealed a main effect of context (see Figure 2): participants responded faster to repeated relative to nonrepeated displays (873 vs. 891 ms; *F*[1, 42] = 5.68, *p* = 0.022, $\eta_{p}^{2}=0.12$). The main effect of epoch was also significant, showing that RTs decreased with increasing epochs (epoch 1 = 911 ms, epoch 4 = 862 ms; *F*[3, 126] = 16.94, *p* < 0.001, $\eta_{p}^{2}=0.29$). Moreover, there was a reliable context × epoch interaction (*F*[3, 126] = 6.36, *p* < 0.001, $\eta_{p}^{2}=0.13$), indicating that contextual cueing, that is, difference in mean RTs between repeated and nonrepeated displays, was measurable from epoch 2 onward (epoch 1: mean contextual-cueing effect: −9 ms; *F*[1, 42] = 0.65, *p* = 0.424, $\eta_{p}^{2}=0.02$; epochs 2–4: mean contextual-cueing effect: 27 ms, all *p*s < 0.05).

Of theoretical interest, there was a significant search mode × phase × context interaction, *F*(1, 42) = 6.42, *p* = 0.015, $\eta_{p}^{2}=0.13$, as well as a search mode × phase × context × epoch interaction (*F*[3, 126] = 3.33, *p* = 0.022, $\eta_{p}^{2}=0.07$), indicating differential relearning effects between the two attention conditions. Exploring the latter interaction further revealed that for the *distributed attention condition,* there was a significant context x phase interaction (*F*[1, 21] = 5.65, *p* = 0.027, $\eta_{p}^{2}=0.21$). During the learning phase, participants responded faster to repeated displays relative to nonrepeated displays (807 vs. 835 ms; main effect of context: *F*[1, 21] = 7.9, *p* = 0.01, $\eta_{p}^{2}=0.27$). We also observed a significant context × epoch interaction in this phase (*F*[3, 63] = 3.23, *p* = 0.028, $\eta_{p}^{2}=0.13$), indicative of the fact that learning about repeated configurations was stable from epoch 2 onward (all *p*s < 0.05). These results imply that contextual cueing developed over time and was the strongest in the last epoch of the learning session (see Figure 2). For the test session, we found a significant main effect of context: there was a 42-ms reaction time benefit for repeated compared to non-repeated displays (*F*[1, 21] = 17.11, *p* < 0.001, $\eta_{p}^{2}=0.45$). Of note, the context × epoch interaction was nonsignificant in this phase (*F*[3, 63] = 2.11, *p* = 0.107, $\eta_{p}^{2}=0.09$). This shows that with distributed attention, participants were able to quickly incorporate the changed target position into the existing context representation, as evident by the reliable—and rapid onset of the—contextual-cueing effect throughout the test phase, after target relocation.

In the *focused attention condition,* the context × phase × epoch interaction was significant (*F*[3, 63] = 3.01, *p* = 0.037, $\eta_{p}^{2}=0.13$). As shown in Figure 2, contextual cueing increased with increasing epochs in the learning phase, a finding that was substantiated by a (marginally) significant context × epoch interaction (*F*[3, 63] = 2.7, *p* = 0.053, $\eta_{p}^{2}=0.11$): there was a clear RT benefit for repeated over nonrepeated displays in epoch 4 compared with epoch 1 (*F*[1, 21] = 11.09, *p* = 0.003, $\eta_{p}^{2}=0.35$). In the test phase, the context × epoch interaction was significant (*F*[3, 63] = 7.94, *p* < 0.001, $\eta_{p}^{2}=0.27$), revealing directly after relocation significant negative cueing effects of −43 ms and −24 ms in epoch 5 (*t*[21] = 2.95, *p* < 0.01), and epoch 6 (t[21] = 1.71, *p* > 0.1), respectively. Subsequently, in epoch 7, a positive cueing effect of 38 ms (*t*[21] = −2.31, *p* = 0.03) emerged, but this difference was not reliable again in epoch 8 (10 ms; *t*[21] = −0.61, *p* > 0.5). These results indicate that while participants developed a contextual-cueing effect gradually over the learning phase, with focused attention the cueing effect recovered only slowly (and was unstable) after target relocation in the test phase.

In a final analysis, we compared contextual cueing between the distributed and focused conditions in each phase. For the learning phase, contextual cueing was found to be comparable between the two attention conditions (all interactions that involved the factor search mode were non-significant, all *p*s > 0.6). But the results were different for the test phase, in which contextual cueing was reduced with focused relative to distributed attention (−5 ms and 43 ms, respectively; significant search mode × context interaction: *F*[1, 42] = 10.23, *p* = 0.003, $\eta_{p}^{2}=0.2$). This result indicates that after a change of the target location, the adaptation of contextual cueing was more pronounced when attention was distributed (as compared to focused). No other main effects or interactions reached significance.

### Control analysis

**Figure 3.**
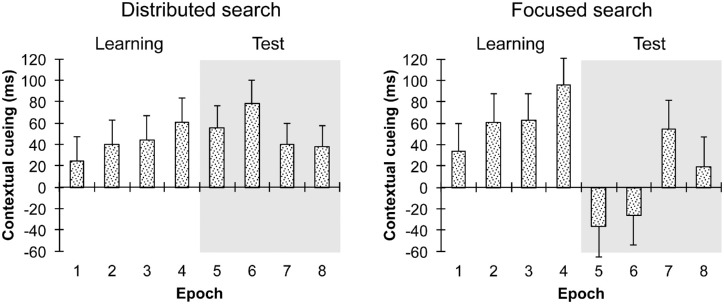
Mean contextual-cueing effects (in ms) in the distributed attention and focused attention conditions (left and right panels, respectively) in each epoch, plotted for the sub-group of 37 participants who showed cueing effects in the learning phase.

An additional analysis was performed to rule out an alternative account of “learning speed.” For instance, it is possible that the two groups differed with regard to the number of observers who showed a cueing effect initially, during the learning phase. Previous studies found that not all observers in search studies develop a contextual-cueing effect (e.g., Lleras & von Mühlenen, 2004). Applied to context adaptation this could mean that observers who fail to exhibit contextual cueing within the initial learning phase are likely to acquire memory of the target in relation to the invariant distractor configuration only later on, that is, they would only show successful learning of relocated targets in the test phase (see, e.g., Zellin et al., 2013a). The current data would be agnostic regarding whether observers in the distributed and focused attention groups differed in their ability for context adaptation or whether there were “only” between-group differences in terms of the observers’ initial speed of learning. To address this issue, we reanalyzed contextual cueing in distributed versus focused search during the learning and test phases and now only considered observers with positive (above zero) cueing effects during initial learning (this criterion led to the removal of seven participants in the distributed attention and eight participants in the focused attention conditions). With this criterion, we effectively removed all participants who did not show contextual learning initially, and the resulting analysis thus provides an unconfounded measure of context adaptation. In this subset analysis, we found a result pattern that was overall comparable to the analysis of the complete sample (see above and Figure 3). Direct tests (based on a significant search mode × phase × context × epoch interaction: *F*[3, 87] = 3.09, *p* = 0.031, $\eta_{p}^{2}=0.1$) revealed a significant cueing effect of 39 ms and 53 ms in the learning and test phases (*t*[15] = −5.16, *p* < 0.001, and *t*[15] = −4.68, *p* < 0.001), respectively, which amounts to an overall cueing effect across the two phases (main effect of context: *F*[1, 15] = 37.73, *p* < 0.001, $\eta_{p}^{2}=0.72$), with the *distributed search mode*. This shows that contextual cueing was stable, exhibiting a reliable benefit across the two phases in this condition. A contextual-cueing effect was also evident with a *focused search mode*, but it occurred only during the learning phase (63 ms, *t*[14] = −5.77, *p* < 0.001) and not during the test phase (3 ms; *t*[14] = −0.22, *p* > 0.8), confirming a lack-of-adaptation effect in this condition. In the respective learning phases, the cueing effect did not differ between the two search modes, as evidenced by nonsignificant search mode × context (*F*[1, 29] = 2.35, *p* = 0.136, $\eta_{p}^{2}=0.08$) and search mode × context × epoch interactions (*F*[3, 87] = 0.21, *p* = 0.891, $\eta_{p}^{2}=0.01$) in this phase. Together, these results show that the overall pattern of contextual cueing during learning and test was largely comparable for the complete sample of observers, as well as for the selection of observers who showed reliable contextual cueing initially. This indicates that variations in the speed of learning cannot account for the difference in contextual adaptation between the distributed and focused search modes.

### Eye movements

**Figure 4.**
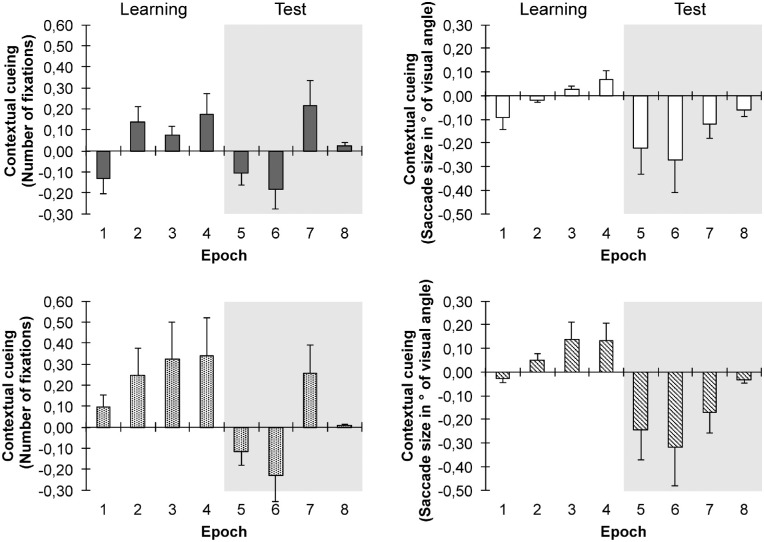
Analyses of eye movements in the focused attention condition: contextual-cueing effects (i.e., gaze measure in non-repeated minus repeated displays) are shown for mean saccade amplitudes and mean number of fixations (left and right panels, respectively) as a function of epoch in the learning and test phases and separately for the entire sample of 26 participants (top panels) or for a subset of 18 observers who showed contextual cueing initially during learning (bottom panels). Error bars: mean standard errors.

We also analyzed the pattern of fixations in the focused attention condition (see Peterson & Kramer, 2001; Tseng & Li, 2004; Zang, Jia, Müller, & Shi, 2015; Kröll et al., 2019). This analysis revealed a significant context × phase × epoch interaction, *F*(3, 57) = 3.84, *p* = 0.014, $\eta_{p}^{2}=0.17$, mirroring the RT pattern of performance. By the last epoch of the learning phase, the mean number of fixations required to find the target was smaller for repeated than for non-repeated displays (4.79 vs. 4.96; t[19] = −1.92, *p* < 0.05; epochs 1–3: *p*s > 0.05); by contrast, in the test phase, the number of fixations was overall comparable between both types of displays (4.92 vs. 4.91; *F*[1, 19] = 0.05, *p* = 0.832, $\eta_{p}^{2}=0$; see Figure 4, top-left panel). An additional analysis of the saccade amplitudes (Figure 4, top-right panel) also revealed a marginally significant context × phase interaction (*F*[1, 19] = 3.08, *p* = 0.095, $\eta_{p}^{2}=0.14$): in the learning phase, the saccade amplitudes were comparable for repeated vs. non-repeated displays (3.32 vs. 3.31; *t*[19] = 0.07, *p* > 0.9), but after the change of the target location in the test phase, the saccade amplitudes became larger for repeated relative to nonrepeated arrays (3.48 vs. 3.31; t[19] = 2.82, *p* < 0.01). The increase in mean saccade amplitudes after target location changes (in nevertheless constant distractor arrays) may index incorrect fixations toward the initially learned—but now changed—target positions (see Pollmann & Manginelli, 2009). Note also that the pattern of results was again very similar for the complete sample observers and for the sample of observers that in particular showed reliable contextual cueing during learning (see Figure 4 – bottom panels). Overall, these findings suggest that eye-movement parameters provide a reliable index for the overall acquisition of context-based memories. The sensitivity of eye-movement measures to contextual cueing was also revealed by significant correlations between the mean RT cueing effect and oculomotor variables: correlations between the mean RT cueing effect and the mean number of fixations were significant in the learning phase (*r* = 0.88, *p* < 0.00001), but not in the test phase (*r* = 0.08, *p* > 0.4). Likewise, there was a significant correlation between the mean RT cueing effect and the mean saccade amplitude in the learning phase (*r* = 0.35, *p* < 0.002), which was not evident in the test phase (*r* = 0.17, *p* > 0.1). This again shows that the pattern of contextual cueing in RTs across learning and test was also reflected in corresponding eye movement measures.

### Recognition test

To test whether participants gained explicit knowledge, performance in the final recognition test compared the observers’ hit rates (repeated display correctly identified as repeated) with their corresponding false-alarm rates (nonrepeated display incorrectly judged as repeated) by means of a 2 × 2 mixed-design ANOVA with the within-subject factor response type (hit, false alarm) and the between-subject factor search mode (distributed, focused attention). This analysis showed no significant difference between hits and false alarms (49.1% vs. 45.3%; *F*[1, 42] = 1.89, *p* = 0.177, $\eta_{p}^{2}=0.04$), no main effect of search mode (*F*[1, 42] = 3.13, *p* = 0.084, $\eta_{p}^{2}=0.07$), and no interaction between response type and search mode (*F*[1, 42) = 0.94, *p* = 0.339, $\eta_{p}^{2}=0.02$). We also found no significant correlation between participants’ individual contextual-cueing effects and their recognition scores (*r* = −0.21, *t*[42] = −1.39, *p* > 0.15). These results indicate that participants were not able to reliably tell apart repeated from nonrepeated displays.

## Discussion

This study examined whether distributed versus focused attention modulates the degree of adaptation in contextual cueing, that is, observers’ ability to incorporate a change of the target position in a nevertheless constant distractor array into the underlying context memory representation. In the distributed attention condition, participants had to maintain gaze at a central fixation cross while performing the visual search task. Thus the detection and subsequent discrimination of the target could be accomplished only with peripheral vision. In contrast, in the focused attention condition, participants were allowed to move their eyes freely while performing the search task, thus permitting a “serial” scanning of the display. In both conditions, half of the trials contained repeated displays, permitting participants to acquire context-based memories of the target locations. Halfway through the experiment, the target swapped its location with a distractor (while keeping all other repeated distractor locations unchanged) to examine how efficiently the location change could be incorporated into context memories when these were acquired under distributed versus focused attention modes.

### Distributed search facilitates context adaptation

The results showed that repeated exposure to invariant display layouts led to expedited RTs (relative to nonrepeated displays), with contextual cueing being comparable in magnitude between the distributed and focused attention conditions in the initial learning phase. However, while initial context learning was equally efficient, in the subsequent test phase, the ability to incorporate the target position change into the existing context representation differed substantially between the two conditions. First, when a distributed attention mode was enforced, the adaptation of contextual cueing was relatively efficient, as evidenced by a rapid and reliable recovery of the contextual-cueing effect within the first two epochs after target relocation. Second, the focused attention mode, by contrast, failed to show a significant contextual-cueing effect in epochs 5 and 6 of the test phase, that is, there was no evidence for an effective adaptation of previously acquired contextual memories. Instead, the change of the target location led to a transient reduction of the contextual-cueing effect immediately after the change (i.e., relative to the last epoch before the change, revealing even an RT cost associated with repeated contexts; see also Makovski & Jiang, 2010; Conci et al., 2011) and the cueing effect did not reliably recover with extended practice with the relocated displays. Of note, a near-identical pattern of results was revealed in an analysis that only included observers who showed a contextual-cueing effect during initial learning. Thus this indicates that the efficient adaptation in the distributed attention mode was not simply due to some observers exhibiting “late learning” (Zellin et al., 2013a). Although this pattern essentially replicates previous studies, which also failed to find effective contextual adaptation (e.g., Annac et al., 2017; Zellin et al., 2014), the examination of observers’ oculomotor behavior yielded some additional, new insights into the processes of context learning and adaptation: successful contextual learning was characterized by fewer fixations and shorter saccades, while adaptation to the relocated target again resulted in longer saccades. Furthermore, the magnitude of the RT cueing effect was significantly correlated with fixation number (and saccade amplitude). Interestingly, in the test phase, when the repeated context was no longer predictive of the initial target position, the context-related gains as evident in the number of fixations and in the length of saccades were effectively abolished. In fact, there were more (and longer) saccades required until the relocated target within a repeated display was detected, relative to a target in a non-repeated display. This impairment of search in repeated relative to novel displays may arise because repeated display arrays continue to “trigger” the initially traversed and consolidated (“learnt”) oculomotor scan-paths (Tseng & Li, 2004; Manginelli & Pollmann, 2009; Kröll et al., 2019) after the change, thus misguiding attention toward the original target position.

In general, these results are consistent with previous reports on the distinction between more *distributed* or more *focused* search modes of visual attention (Treisman, 2006). Treisman referred to distributed attention in terms of a relatively broad attentional focus that allows a rapid extraction of the global gist of a scene (Thorpe, Fize, & Marlot, 1996; Rosenholtz, Huang, Raj, Balas, & Ilie, 2012) and, along with it, the statistical regularities in the environment (Chong & Treisman, 2003; Chong, Joo, Emmanouil, & Treisman, 2008). Importantly, it has been argued that the scene gist provides information about the spatial layout of the search items (for a review see Wolfe et al., 2011). Additionally, it has been shown that peripheral vision alone is sufficient for observers to extract the overall gist for categorizing a scene (Li, VanRullen, Koch, & Perona, 2002). In turn, narrowing attention down to individual items (Posner, 1980; Treisman & Gelade, 1980) serves to process (and integrate) the detailed features of a single object at a given location. In line with these findings, here we show that inducing one or the other processing mode in turn leads to variations in the flexibility of context adaptation. Specifically, distributed attention appears to facilitate processing of the whole spatial array, thus providing a context representation that is rather flexible and which can be updated readily. By contrast, when attention is focused, which is the typical, “default” processing mode in search tasks that require close scrutiny of individual items (as in the present letter search task), then changes of the target location are not easily integrated in the existing context memory representation, as evidenced by a lack of contextual adaptation (see also Annac et al., 2017; Makovski & Jiang, 2010; Manginelli & Pollmann, 2009; Zellin et al., 2013a). However, this does not mean that there is no context adaptation with focused search at all. Instead, relearning should eventually take place and this is what has actually been demonstrated in a previous study where we tested the adaptation to a change in the longer term (Zellin et al., 2014).

Our finding is also broadly consistent with the study of Lleras and von Mühlenen (2004), who asked two groups of participants to perform a contextual cueing task using different search strategies. In the active strategy group, participants were instructed to search through the display “as actively as possible,” whereas in the passive strategy group, participants were instructed to “be as receptive as possible” when searching for the target. Contextual cueing effects were found to be more marked in the passive than in the active search condition, which was taken to indicate that passive, that is, more broadly distributed, search may facilitate access to implicitly learnt context information, thereby improving search performance. Our results essentially support these findings, when assuming that a “passive search” set goes along with a wider spatial tuning of the attentional window within which a peripherally located stimulus can be detected: more broadly distributed search may facilitate access to implicitly learnt context information, which in turn improves search performance. Note, though, the results of the current eye-tracking study go beyond those of Lleras and von Mühlenen in demonstrating that peripheral processing of repeated search layouts particularly facilitates the adaptation of acquired context memories after target location changes.

### Global and local contextual cueing

A number of contextual cueing studies suggest that it is predominantly the spatial configuration of distractors in the immediate vicinity of the target that brings about the cueing effect (Brady & Chun, 2007; Olson & Chun, 2002). Why, then, would a distributed attentional mode specifically facilitate context adaptation? A possible explanation might be that distributed attention particularly promotes the formation of global target-distractor representations (i.e., across a larger region of space). Indeed, there is evidence that contextual cueing is also supported by memory of the entire distractor configuration, including (not only distractor-target, but also) distractor-distractor associations (Beesley et al., 2015; Jiang & Wagner, 2004). The formation of such a global representation under a distributed search mode might in turn promote the inhibition of the repeating distractors (see Ogawa, Takeda, & Kumada, 2007) thus facilitating target adaptation, as compared to a more prevailing facilitation of the target with a more focused search mode, which would be less flexible to incorporate a change. Moreover, within such a “global” representation, target location changes would require “only” a single change of the underlying representation: that of the changed target position in relation to the invariant distractor configuration, while leaving associations among individual distractor locations unaffected. That is, the global distractor representation would still provide a reliable contextual cue for the changed target position, thus enabling observers to form a new association between the changed target position and the constant distractor context in the test phase.

This proposal is consistent with Henke's (2010) notion of functionally different memory systems responsible for flexible episodic memory representations (in hippocampus) and, respectively, more rigid, “unitized” (parahipocampal) memory representations. Interestingly, both of these anatomical structures have been reported in previous Functional magnetic resonance imaging (fMRI) investigations of contextual cueing (e.g., Preston & Gabrielli, 2008; Greene, Gross, Elsinger, & Rao, 2007; Westerberg, Miller, Reber, Cohen, & Paller, 2011; Geyer, Baumgartner, Müller, & Pollmann, 2012). Thus, distributed processing might activate flexible hippocampus-based memory representations that are easier to adapt to target location changes, while focused search activates more rigid, parahippocampus-based representations that are more impervious to incorporating such changes.

### Corroboration of the “dissociation” between focused and distributed modes of search

The present pattern of findings receives support from the extant contextual-cueing literature (focused search mode condition) and, respectively, a replication of the results from the distributed search mode condition. Concerning the former condition, a number of previous studies, all using the current “learning-phase/test-phase” design, had already found the adaptability of context memory to be severely limited (e.g., Manginelli & Pollmann, 2009; Makovski & Jiang, 2010; a representative meta-analytical data set with 85 participants is provided in Annac et al., 2017). Collectively, these studies report that contextual facilitation drops massively following target location changes at the transition from the learning to the test phase. Of note, in all of these studies, observers performed the search tasks under unrestricted, free-viewing conditions. Given the relative difficulty of the “T” versus “L” form-conjunction search task (which affords some, but very little bottom-up guidance; see Moran, Zehetleitner, Müller, & Usher, 2013), search would have been performed in “focused” mode, that is focal attention, and the eye (e.g., Peterson & Kramer, 2001; Tseng & Li, 2004; Zang et al., 2015, Manginelli & Pollmann, 2009), had to be shifted to various display regions until (relevant “local” context cues were picked up and) the target was eventually detected. The results from the present “focused-attention” condition perfectly match the prior findings: there is little recovery of cueing after target position changes when the (repeated) search arrays were originally trained in “focused” mode.

Because there are (to our knowledge) no prior studies that tested whether changes can be adapted more flexibly under a “distributed-attention” mode, we ran a replication experiment for this condition, with participants having to maintain fixation in the display center. There were 11 new participants in the replication experiment, all with an above-zero contextual-cueing effect in the initial learning phase. The results revealed a significant main effect of context (*F*[1, 10] = 30.82, *p* < 0.001, $\eta_{p}^{2}=0.75$), thus showing a reliable contextual-cueing effect overall. Importantly, the context by phase interaction was not significant (*F*[1, 10] = 0.29, *p* = 0.599, $\eta_{p}^{2}=0.03$), and contextual cueing was evident in both the learning phase (*F*[1, 10] = 21.73, *p* = 0.001, $\eta_{p}^{2}=0.68$) and the test phase (*F*[1, 10] = 6.83, *p* = 0.026, $\eta_{p}^{2}=0.41$): the contextual facilitation remained as large (numerically and statistically) in the test phase, after target relocation, as in the preceding training phase, with the original target location: 39 versus 53 ms, respectively. This confirms that, when repeated displays are learnt in “distributed-attention” mode, contextual guidance of visual search can effectively adapt to the target relocation in the test phase; in other words, training in distributed, “global” mode allows the new target location to be effectively incorporated in the search-guiding memory.

## Conclusion

Learnt target-distractor contexts guide visual search. However, updating a previously acquired target-distractor memory subsequent to a change of the target location has been found to be rather inefficient and slow. These results show that the imperviousness of contextual memory to incorporating relocated targets is particularly pronounced when observers adopt a narrow focus of attention to perform a rather difficult form-conjunction search task. By contrast, when they adopt a broad attentional distribution, context-based memories can be updated more readily because this mode promotes the acquisition of more global contextual representations that continue to provide effective cues even after target relocation.
